# The Distribution of Major Brain Metabolites in Normal Adults: Short Echo Time Whole-Brain MR Spectroscopic Imaging Findings

**DOI:** 10.3390/metabo12060543

**Published:** 2022-06-14

**Authors:** Xinnan Li, Kagari Abiko, Sulaiman Sheriff, Andrew A. Maudsley, Yuta Urushibata, Sinyeob Ahn, Khin Khin Tha

**Affiliations:** 1 Laboratory for Biomarker Imaging Science, Hokkaido University Graduate School of Biomedical Science and Engineering, Sapporo 060-8638, Japan; lixinnan820@yahoo.co.jp; 2 Department of Rehabilitation, Hokkaido University Hospital, Sapporo 060-8648, Japan; kagari.abiko@gmail.com; 3 Department of Rehabilitation, Sapporo Azabu Neurosurgical Hospital, Sapporo 065-0022, Japan; 4 Department of Radiology, University of Miami School of Medicine, Miami, FL 33146, USA; ssheriff@med.miami.edu (S.S.); amaudsley@med.miami.edu (A.A.M.); 5 Siemens Healthcare K.K., Tokyo 141-8644, Japan; yuta.urushibata@siemens-healthineers.com; 6 Siemens Healthineers, San Francisco, CA 94553, USA; sinyeob.ahn@siemens-healthineers.com; 7 Global Center for Biomedical Science and Engineering, Hokkaido University Faculty of Medicine, Sapporo 060-8638, Japan

**Keywords:** echo-planar, magnetic resonance spectroscopic imaging, metabolite, whole-brain

## Abstract

This prospective study aimed to evaluate the variation in magnetic resonance spectroscopic imaging (MRSI)-observed brain metabolite concentrations according to anatomical location, sex, and age, and the relationships among regional metabolite distributions, using short echo time (TE) whole-brain MRSI (WB-MRSI). Thirty-eight healthy participants underwent short TE WB-MRSI. The major metabolite ratios, i.e., N-acetyl aspartate (NAA)/creatine (Cr), choline (Cho)/Cr, glutamate + glutamine (Glx)/Cr, and myoinositol (mI)/Cr, were calculated voxel-by-voxel. Their variations according to anatomical regions, sex, and age, and their relationship to each other were evaluated by using repeated-measures analysis of variance, t-tests, and Pearson’s product-moment correlation analyses. All four metabolite ratios exhibited widespread regional variation across the cerebral hemispheres (corrected *p* < 0.05). Laterality between the two sides and sex-related variation were also shown (*p* < 0.05). In several regions, NAA/Cr and Glx/Cr decreased and mI/Cr increased with age (corrected *p* < 0.05). There was a moderate positive correlation between NAA/Cr and mI/Cr in the insular lobe and thalamus and between Glx/Cr and mI/Cr in the parietal lobe (r ≥ 0.348, corrected *p* ≤ 0.025). These observations demand age- and sex- specific regional reference values in interpreting these metabolites, and they may facilitate the understanding of glial-neuronal interactions in maintaining homeostasis.

## 1. Introduction

Magnetic resonance spectroscopy (MRS) has been applied in clinical practice for several decades for in vivo measurement of metabolites that cannot be directly detected by routine MR imaging sequences [1]. Its usefulness in diagnosing brain diseases or monitoring treatment has been widely documented; for example, malignant brain tumors are characterized by increased choline (Cho) and decreased N-acetylaspartate (NAA) signals, relative to normal tissue [1], and increased Lactate (Lac) concentration has been used as an indicator of disease activity in mitochondrial diseases, such as Leigh syndrome, mitochondrial encephalomyopathy, lactic acidosis, and stroke-like episodes (MELAS) [2,3].

Despite its clinical usefulness, the widespread application of MRS for clinical studies remains limited, for reasons that include the limited spatial sampling and long acquisition times provided by the standard single voxel acquisition methods [1]. Several documents have reported on MR spectroscopic imaging (MRSI) techniques that enable mapping of tissue metabolites over larger brain regions within clinically acceptable acquisition times [4,5]. Of these, an echo-planar spectroscopic imaging method has recently emerged as a whole-brain acquisition (WB-MRSI) in time suitable for clinical studies [6,7]. The reproducibility of WB-MRSI has been reported as comparable to that of the conventional MRS technique [8]. Studies using WB-MRSI have also reported its ability to detect metabolite alterations in the normal-appearing brain regions of patients with traumatic brain injury, amyotrophic lateral sclerosis, and brain tumors, suggestive of its high sensitivity in detecting abnormalities across the whole brain, which is not possible with single-voxel spectroscopy [9,10,11].

Identification of metabolite alterations by MRS requires knowledge about normal metabolite concentrations and their distribution. Conventional MRS and a few initial WB-MRSI studies have reported variations in metabolite concentrations with anatomical location, sex, age, and echo time (TE) [7,12,13]. The existing WB-MRSI reports that evaluated the variation in metabolite concentrations with anatomical location focus mainly on laterality. Very little has been reported about their variation across various anatomical regions of the brain. To our knowledge, there has been only one WB-MRSI report which statistically compared the concentrations of glutamate and glutamine compounds (Glx) across anatomical regions [14]. With regard to sex-related variations, there have been two reports that compared the major metabolite concentrations and their ratios, using an intermediate TE [7,12]; however, short TE is generally preferred to longer TE settings due to its higher signal-to-noise ratio (SNR) and its ability to detect more metabolites [14].

This prospective study aimed to evaluate the influence of anatomical location, sex, and age on the concentrations of the brain’s major metabolites and their relationship, using short TE WB-MRSI.

## 2. Results

**Figure 1:**
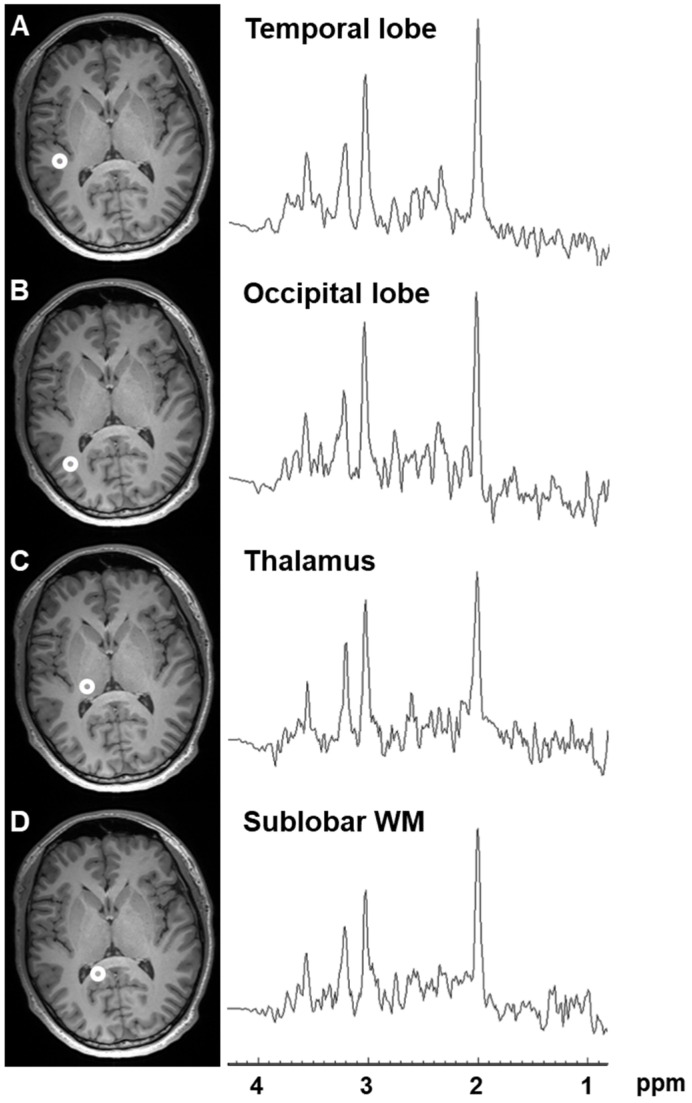
The representative single-voxel spectra obtained from the left (**A**) temporal lobe, (**B**) occipital lobe, (**C**) thalamus, and (**D**) sublobar white matter of WB-MRSI of a 25-year-old male. The voxel locations are shown on the T1-weighted 3D magnetization-prepared rapid acquisition gradient echo (MPRAGE) images.

The representative single-voxel spectra extracted from WB-MRSI and major metabolite ratio maps are shown in Figure 1 and Supplementary Figure S1, respectively. The average Cramer-Rao bound was 9.70 ± 2.45% for NAA, 12.28 ± 2.47% for Cho, 20.20 ± 3.20% for Glx, 12.86 ± 2.04% for mI, and 8.31 ± 2.14% for creatine (Cr).

### 2.1. Variation in Metabolite Ratios with Anatomical Location

**Figure 2:**
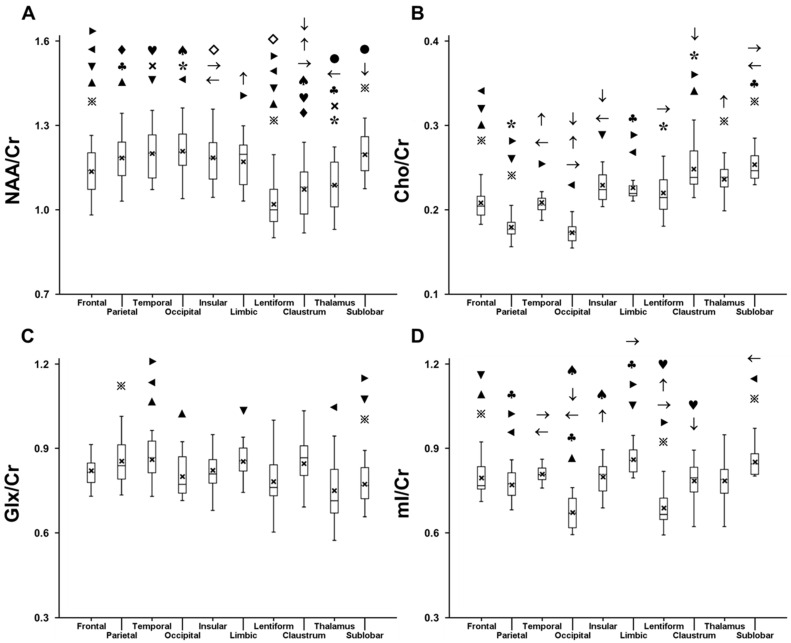
Box and whisker plots showing the region-specific mean (cross mark), median, interquartile range, minimum, and maximum for (**A**) N-acetyl acetylaspartate (NAA)/creatine (Cr), (**B**) choline (Cho)/Cr, (**C**) glutamate + glutamine (Glx)/Cr, and (**D**) myoinositol (mI)/Cr (Abbreviations: frontal = frontal lobe, parietal = parietal lobe, temporal = temporal lobe, occipital = occipital lobe, insula = insular lobe, limbic = limbic lobe, lentiform = lentiform nucleus, sublobar = sublobar white matter). Each symbol indicates pairs with statistical significance (i.e., *p* < 0.05 following correction for multiple comparisons). For example, as indicated by ▶, NAA/Cr is different among the frontal lobe, limbic lobe, and lentiform nucleus.

The regional metabolite ratios are summarized in Figure 2. The metabolite ratios varied significantly among several anatomical locations. The frontal lobe, lentiform nucleus, claustrum, and thalamus had lower NAA/Cr than the parietal, temporal, and occipital lobes and the sublobar white matter (corrected *p* < 0.018). The frontal lobe and claustrum also had lower NAA/Cr than the limbic lobe (corrected *p* < 0.016). The claustrum and thalamus had lower NAA/Cr than the insular lobe (corrected *p* < 0.004). The lentiform nucleus had lower NAA/Cr than the frontal, insular, and limbic lobes (corrected *p* < 0.001). On the other hand, the insular and limbic lobes, claustrum, thalamus, and the sublobar white matter had higher Cho/Cr than the four major cerebral lobes (corrected *p* < 0.001). The frontal and temporal lobes and lentiform nucleus had higher Cho/Cr than the parietal and occipital lobes (corrected *p* < 0.001). The claustrum and sublobar white matter had higher Cho/Cr than the insular and limbic lobes and lentiform nucleus (corrected *p* < 0.001). The sublobar white matter also had higher Cho/Cr than the thalamus (corrected *p* = 0.017) and had lower Glx/Cr than the parietal, temporal, and limbic lobes (corrected *p* < 0.015). The occipital lobe and thalamus had lower Glx/Cr than the temporal lobe (corrected *p* < 0.023). The limbic lobe and sublobar white matter had higher mI/Cr than all four major cerebral lobes and lentiform nucleus (corrected *p* < 0.015). The frontal, parietal, temporal, and insular lobes had higher mI/Cr than the occipital lobe and lentiform nucleus (corrected *p* < 0.006). The claustrum also had higher mI/Cr than the occipital lobe and lentiform nucleus (corrected *p* < 0.048).

The gray and white matter volume ratios and their standard deviations (SD) are: 0.771 ± 0.077 for frontal lobe, 1.282 ± 0.128 for parietal lobe, 1.152 ± 0.090 for temporal lobe, 1.340 ± 0.137 for occipital lobe, 1.260 ± 0.253 for insular lobe, 1.044 ± 0.113 for limbic lobe, 1.082 ± 0.184 for claustrum, 1.925 ± 0.722 for lentiform nucleus, 0.898 ± 0.153 for thalamus, and 0.356 ± 0.079 for sublobar white matter. Tissue-specific regional metabolite ratios are provided in Supplementary Table S1.

### 2.2. Laterality

The mean regional metabolite ratios of anatomical regions by each side are provided in Table 1. The left frontal (*p* = 0.028), parietal (*p* = 0.002), and temporal (*p* < 0.001) lobes had significantly higher NAA/Cr than the right side. The left frontal (*p* < 0.001), temporal (*p* < 0.001), and limbic (*p* = 0.010) lobes, claustrum (*p* = 0.034), and the sublobar white matter (*p* = 0.002) had significantly higher Glx/Cr than the right side. The right parietal (*p* < 0.001), temporal (*p* < 0.001), occipital (*p* < 0.001), insular (*p* = 0.024) and limbic (*p* = 0.004) lobes, and the sublobar white matter (*p* < 0.001) had significantly higher Cho/Cr than the left side. No laterality in mI/Cr was observed for any anatomical region. A separate evaluation with 30 right-handed participants also gave similar results for most anatomical regions (Supplementary Table S2).

### 2.3. Sex-Related Variation

The mean regional metabolite ratios of each sex group are provided in Table 1. Women had significantly lower Cho/Cr in the frontal (*p* = 0.009), temporal (*p* = 0.018), and insular (*p* = 0.012) lobes, and higher mI/Cr in the parietal (*p* = 0.016) and occipital (*p* = 0.005) lobes, and the lentiform nucleus (*p* = 0.025) than men. The sex difference in the metabolite ratios was greatest in the lentiform nucleus for mI/Cr, amounting to 20.3% difference.

The sex-related variation mentioned above was also true for mI/Cr in the left occipital lobe (*p* = 0.003) and the lentiform nucleus (*p* = 0.044) if the metabolite ratios were compared for each side (Supplementary Table S3). For the right cerebral hemisphere, no significant sex difference in mI/Cr was observed. As for Cho/Cr, women showed a trend toward a decrease in the right frontal (*p* = 0.051), left temporal (*p* = 0.063), and left insular (*p* = 0.053) lobes.

### 2.4. Correlation with Age

Table 2 summarizes the results of correlation of each metabolite ratio with age. NAA/Cr significantly decreased with age in most anatomical locations, including the frontal (r = −0.741, corrected *p* < 0.001), parietal (r = −0.791, corrected *p* < 0.001), temporal (r = −0.819, corrected *p* < 0.001), occipital (r = −0.668, corrected *p* < 0.001), insular (r = −0.605, corrected *p* < 0.001) and limbic (r = −0.780, corrected *p* < 0.001) lobes, claustrum (r = −0.512, corrected *p* = 0.003), thalamus (r = −0.471, corrected *p* = 0.005), and the sublobar white matter (r = −0.568, corrected *p* < 0.001). Glx/Cr also significantly decreased with age in the parietal (r = −0.532, corrected *p* < 0.001) and limbic (r = −0.422, corrected *p* = 0.011) lobes. A significant increase in mI/Cr with age was observed in the frontal (r = 0.417, corrected *p* = 0.045), insular (r = 0.452, corrected *p* = 0.006), and limbic (r = 0.385, corrected *p* = 0.040) lobes.

The above observations were also valid for each side of the anatomical regions that exhibit laterality in the metabolite ratios, except the NAA/Cr of bilateral claustra, left thalamus, sublobar white matter, and the right occipital lobe, the Glx/Cr of bilateral limbic and right parietal lobes, and the mI/Cr of bilateral frontal and limbic lobes, and the left insular lobe. Further exploration of the influence of sex on age-related variation in metabolite ratios also revealed significant differences: a decrease in NAA/Cr in the frontal (r = −0.794, corrected *p* < 0.001), parietal (r = −0.783, corrected *p* < 0.001), temporal (r = −0.832, corrected *p* < 0.001), and limbic (r = −0.834, corrected *p* < 0.001) lobes was observed in women. The men’s results were also similar in most locations, except increased mI/Cr in the frontal and limbic lobes.

### 2.5. Relationship among Regional Metabolite Ratios

**Figure 3:**
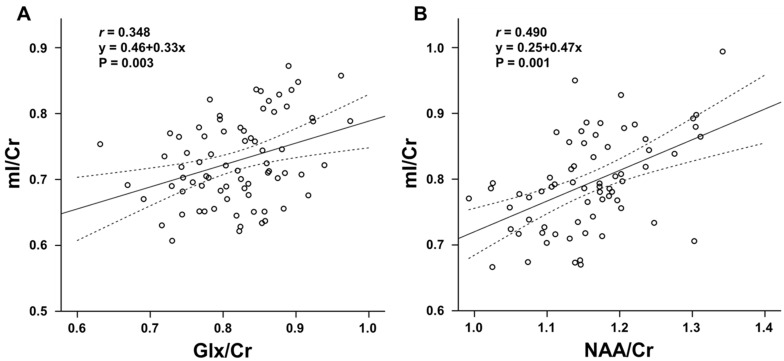
Scatterplots showing the correlation between (**A**) glutamate + glutamine (Glx)/creatine (Cr) and myoinositol (mI)/Cr of the parietal lobe (r = 0.348), (**B**) N-acetylaspartate (NAA)/Cr and mI/Cr of the insular lobe (r = 0.490). The straight line indicates the mean and the dotted lines 95% confidence interval. These correlations are statistically significant at corrected *p* < 0.05 (Pearson’s product-moment correlation analyses).

Figure 3 provides a summary of significant relationships among regional metabolite ratios. Moderate positive correlations were observed between NAA/Cr and mI/Cr in the insular lobe (r = 0.490, corrected *p* < 0.001) and thalamus (r = 0.362, corrected *p* = 0.025), and between Glx/Cr and mI/Cr in the parietal lobe (r = 0.348, corrected *p* = 0.018).

When these correlations were tested on each side, moderate positive correlations were observed between NAA/Cr and mI/Cr in the left insular lobe (r = 0.619, corrected *p* = 0.002), and between Glx/Cr and mI/Cr in the left parietal lobe (r = 0.503, corrected *p* = 0.013). When tested on each sex group, moderate positive correlations were observed between NAA/Cr and mI/Cr in the insular lobe (r = 0.642, corrected *p* = 0.001) and the thalamus (r = 0.547, corrected *p* = 0.013), in women.

### 2.6. The Influence of Scan Conditions on Laterality in Major Metabolite Ratios

Bland-Altman plots are summarized in Supplementary Figure S2. For NAA/Cr, Cho/Cr, Glx/Cr, and mI/Cr, the mean differences and their SD were 0.096 ± 0.048, 0.013 ± 0.011, −0.004 ± 0.099, and −0.038 ± 0.112, respectively. The difference in metabolite ratios between the two head positions fell within the 95% confidence intervals, except for NAA/Cr and Glx/Cr in the left lentiform nucleus, Cho/Cr and mI/Cr in the right lentiform nucleus, and mI/Cr in the right thalamus. The laterality pattern was similar across all regions of interest (ROIs) between the two head positions, except for Cho/Cr in the limbic lobe, Glx/Cr in the parietal and temporal lobes, lentiform nucleus, and thalamus, and mI/Cr in the temporal lobe, claustrum, and sublobar white matter.

## 3. Discussion

This report evaluated the influence of anatomical location, sex, and age on the brain’s major metabolite ratios and the relationship among these ratios, using short TE WB-MRSI. Metabolite ratios, instead of absolute metabolite concentrations, were evaluated because metabolite ratios are more commonly used in real clinical situations due to simpler acquisition and data processing [14]. Additionally, evaluating metabolite ratios can reduce the influence of T2 relaxation time [15]. Significant differences in metabolite ratios were observed across anatomical locations, sex, and age, which indicates these must be accounted for when interpreting regional metabolite ratios. There were also correlations among regional metabolite ratios, the knowledge of which facilitates the understanding of glial-neuronal interactions, and the involvement of each metabolite in regulating homeostasis.

NAA/Cr in the deep gray matter nuclei was significantly lower than most other anatomical regions. This metabolite ratio was also significantly lower in the frontal lobe, when compared with the other major anatomical lobes. NAA concentrations have been shown to associate positively with neuronal density [16,17]. Thus, these findings may reflect the lower neuronal density in these regions. Supportive of this, histological studies on cadaveric brains have documented lower neuronal density in these regions [18,19]. Our observation of lower Cho/Cr in the parietal and occipital lobes is in agreement with a previous autopsy report that observed a low concentration of Cho-containing compounds in these regions [20]. The sublobar white matter had relatively higher Cho/Cr, which may be due to a relatively higher Cho and lower Cr in the whiter matter than the gray matter [21]. Given that glutamate is the main component of Glx, Glx/Cr variations are thought to reflect variations in neuron and glutamate receptor density. Glutamate is mainly located in the synaptic vesicles of nerve terminals [22]. Glx/Cr variations in this study replicate the results of a previous report which shows higher Glx/Cr in the temporal lobe than the thalamus and no significant variations among the frontal, parietal, and temporal lobes [23]. Lower mI/Cr in the occipital lobe than the other major cerebral lobes is a new finding of this study. As mI is considered a glial marker, the lower mI/Cr in the occipital lobe and lentiform nucleus may be due to fewer glial cells in these regions, compared to the other major cerebral lobes. Supportive of this observation, a postmortem study has shown fewer astrocytes in the occipital lobe and basal ganglia than the other major cerebral lobes [24]. Noteworthily, a few previous studies have also shown lower prevalence of low-grade gliomas in the occipital lobe [25]. Although the mechanisms underlying this regional variation and glioma development may be more complex, a variation in glial cell population may also be implicated [26].

Evaluation of regional variation in metabolite ratios is incomplete without evaluating laterality. For several anatomical regions, the left side had higher NAA/Cr or Glx/Cr and lower Cho/Cr than the right side. The previous reports have also observed higher NAA/Cr in the left temporal, parietal, and occipital lobes; higher Cho/Cr in the gray matter of the right four major cerebral lobes; and higher Glx in the white matter of the temporal lobe [7,27]. Laterality in Cho/Cr and Glx/Cr in the sublobar white matter and that in Glx/Cr in the frontal and limbic lobes are the new observations of this study. These observations are thought to highlight the importance of such knowledge in diagnosing pathologies, as the unilateral increase in Cho/Cr and glutamate concentration has been reported in demyelinating, inflammatory, or autoimmune diseases [28,29]. Given the high percentage of right-handed participants in this study, these observations may reflect handedness-dependent lateralization in metabolite ratios. Since NAA and Glx reflect neuronal density, higher NAA/Cr and Glx/Cr in the left cerebral hemisphere may be reflective of higher neuronal density in the dominant hemisphere. Aligning with this, previous histological studies on Nissl-stained sections of human adult brains have reported a larger neuronal density in the left Broca’s area and superior frontal gyrus [30,31]. Hemispheric asymmetry in myelin content may have contributed to the observed Cho difference [32]. Thicker myelin sheath of axons in the left temporal lobe than the right side and the right-than-left asymmetry in g-ratio—which is the ratio of the axon’s diameter to that of axon and myelin [33]—have been reported in several white matter fasciculi [34,35]. It is considered unlikely that the observed laterality in metabolite ratios is artifactual, for example due to B0 or B1 field inhomogeneity. However, our investigation of the influence of scan conditions on laterality for the major metabolite ratios indicates that that this effect is small for most anatomical regions.

In several anatomical locations, women had lower Cho/Cr and mI/Cr than men. The observations of lower Cho/Cr in the frontal and temporal gray matter and lower mI in the parietal white matter and basal ganglia in women are in agreement with several previous reports [12,29,36,37]. To our knowledge, lower Cho/Cr in the insular lobe and lower mI/Cr in the occipital lobe in women have not been reported. The difference in Cho concentration may be sex hormone-related. Enhanced cholinergic enzyme activity and higher Cho uptake in the cholinergic neurons have been shown in female rats than male rats [38]. There also revealed an increase in Cho/Cr in women who underwent ovarian suppression [39]. Differences in astrocyte number may be attributable to the difference in mI concentration. A postmortem study has reported fewer astrocytes in the frontal, temporal, parietal, and occipital cortex in women than men [40].

This study also observed an age-related decrease in NAA/Cr, and decreased Glx/Cr or increased mI/Cr in several regions, including the frontal and limbic lobes. NAA/Cr and Glx/Cr alterations are thought to reflect an age-dependent neuronal and axonal loss. Age-related decrease in NAA/Cr and Glx concentrations has been reported in the major cerebral lobes and the parietal lobe, respectively [27]. A stronger degree of correlation of NAA/Cr with age than the other metabolite ratios may suggest that NAA/Cr is a sensitive index for age-related change. Age-related FA decrease in the four major cerebral lobes and sublobar white matter, suggestive of neuronal loss, has been reported by a diffusion tensor imaging (DTI) report [41]. From the observation of a stronger degree of correlation of NAA/Cr of the four major cerebral lobes and insular and limbic lobes than the other locations, it is possible that the predominant age-dependent neuronal and axonal loss occurs in these locations. As for mI, a previous study reported an age-related linear increase in mI concentration in the medial frontal gray matter in healthy volunteers aged 19 to 78 years [42]. Another study that employed immunohistochemical staining of the avidin-biotin complex reported a stable and moderate increase in astrocytic cell number with age in the human mid-frontal cortex [43].

A moderate but significant positive correlation between NAA/Cr and mI/Cr was observed in the insular lobe and thalamus, which is a new finding of this study. In addition, tendencies toward positive relationship were present in several anatomical regions. This relationship between NAA/Cr and mI/Cr may reflect an interaction between neurons and astrocytes. Astrocytes are important to maintain the structural integrity of neurons [44]. Perturbations of this relationship can be linked to disease states such as Alzheimer’s disease. A decrease in NAA/mI and its correlation with dementia test score have been reported in Alzheimer’s disease, according to a 7-year longitudinal study [45]. In addition to NAA/Cr, Glx/Cr also showed a moderate positive correlation with mI/Cr in the parietal lobe. This finding may reflect the importance of astrocytes in regulating neurotransmission through glutamate-glutamine cycling. Astrocytes take up glutamate from neurons and release glutamine to neurons in return [46]. A previous report has shown a positive correlation between Glx and mI in the cerebellar vermis of healthy volunteers [47].

There are several limitations in this study. First, the sample size is relatively small. There could be hidden differences in the major metabolite ratios left undiscovered due to type II error. Second, two of the 40 participants had to be excluded due to motion artifacts probably associated with long TA. Further TA reduction is desired for application in clinical settings. An immediate solution may be reducing the spatial resolution or increasing the parallel imaging acceleration factor. Third, several ROIs failed to meet the desired image quality (i.e., linewidth less than 13 Hz and Cramer-Rao lower bound less than 20% for NAA, Cho, and Cr and 30% for Glx and mI) possibly due to B0 field inhomogeneity, which led to the exclusion of the caudate nucleus, midbrain, pons, and cerebellum from further analyses. Although this study could not provide metabolite quantification for these anatomical regions, the average relative error for the other anatomical regions could be retained as low as 7.46 ± 1.82% for NAA/Cr, 9.99 ± 2.31% for Cho/Cr, 17.41 ± 3.04% for Glx/Cr, and 13.18 ± 3.73% for mI/Cr (Note: The average relative error was calculated from Cramer-Rao bounds and the correlation coefficient between the two metabolite concentrations of concern [48]). The lack of significant correlation between the average relative error and the regional metabolite ratios was also confirmed. Fourth, only metabolite ratios normalized by Cr, instead of absolute metabolite concentrations, were evaluated. As mentioned earlier, evaluating metabolite ratios has certain advantages. However, on rare occasions, the interpretation may be affected by diseases causing Cr changes [49]. Fifth, handedness information was not available in eight participants. This might have prevented us from drawing conclusions about the influence of handedness on laterality. Nevertheless, it is considered that this study provides sufficient information about laterality in right-handed individuals.

## 4. Materials and Methods

### 4.1. Participants

This prospective study was approved by our institutional review board. Written informed consent was obtained from all participants. Forty participants were recruited over a period of 21 months (July 2016 through March 2018). The inclusion criterion was age over 20 years, and the exclusion criteria were contraindications for MR imaging, history of diseases that might affect the central nervous system integrity (e.g., psychiatric diseases, diabetes, hypertension), gross abnormalities and white matter hyperintensities greater than scale 1 on the age-related white matter changes (ARWMC) scale [50], and visible motion artifacts on water-reference spectroscopic images. The review of conventional MR imaging sequences and water-reference spectroscopic images to exclude the abnormalities and artifacts was done by a radiologist with 21 years of experience in neuroimaging. Two volunteers were excluded for visible motion artifacts. Thus, a total of 38 participants (21 men and 17 women; age range = 22–68 years) were eligible for the study. Thirty of them were confirmed right-handed, according to the Edinburgh Handedness Inventory [51]. Information about handedness was not available for the other participants. Details about the participants are shown in Table 3.

### 4.2. MR Imaging

#### 4.2.1. For Evaluation of the Distribution of Major Metabolite Ratios

MR imaging was performed using a 3T scanner (MAGNETOM Prisma, Siemens Healthineers, Erlangen, Germany) and a standard 64-channel head/neck coil. Padding and a fixation device were used to minimize head motion during acquisition.

Short TE WB-MRSI was acquired with a prototype volumetric spin-echo echo-planar spectroscopic imaging (EPSI) sequence, which employed two-dimensional phase encoding and echo-planar readout, frequency-selective water suppression, and lipid inversion nulling. The sequence details are as described in a previous report [52]. Regional saturation bands were also applied to suppress out-of-slice signals from areas such as the sinus and orbits. The sequence included a frequency reference measurement and adjustment that corrects for frequency drift throughout acquisition. The developer-recommended scan parameters were used, which included: repetition time (TR)/TE/inversion time (TI) = 1710/17/198 ms, flip angle (FA) = 73°, sampling of 50 × 50 × 18 k-space points over 280 × 280 ×180 mm^3^, and acquisition time (TA) = 16:49 min. The WB-MRSI acquisition also included a second dataset obtained in an interleaved manner without water suppression and 10° excitation and gradient-echo observation (TE = 3.8 ms). This second dataset provided a water reference signal (i.e., water MRSI) with identical spatial parameters as the metabolite MRSI, for normalization of the metabolite concentrations to institutional units [53].

In addition to WB-MRSI, a T1-weighted 3D magnetization-prepared rapid acquisition gradient echo (MPRAGE) sequence (TR/TE/TI = 1900/2.85/900 ms, FA = 9°, voxel size = 0.9 × 0.9 × 0.9 mm^3^, FOV = 230 × 230 mm^2^, matrix = 256 × 256, the number of excitation (NEX) = 1, TA = 4:50 min) was acquired. An axial fluid-attenuated inversion recovery (FLAIR) imaging sequence (TR/TE/TI = 12,000/115/2800 ms) and an axial proton density-weighted imaging (PDWI) sequence (TR/TE = 4000/12 ms) were also acquired to exclude gross abnormalities and white matter hyperintensities.

#### 4.2.2. Test of the Influence of Scan Conditions on Laterality in Major Metabolite Ratios

To test if scan conditions affect laterality in regional metabolite ratios, short TE WB-MRSI was performed in supine and prone positions in a participant (27-year male). For this purpose, a standard 20-channel head/neck coil was used.

### 4.3. Data Analysis

**Figure 4:**
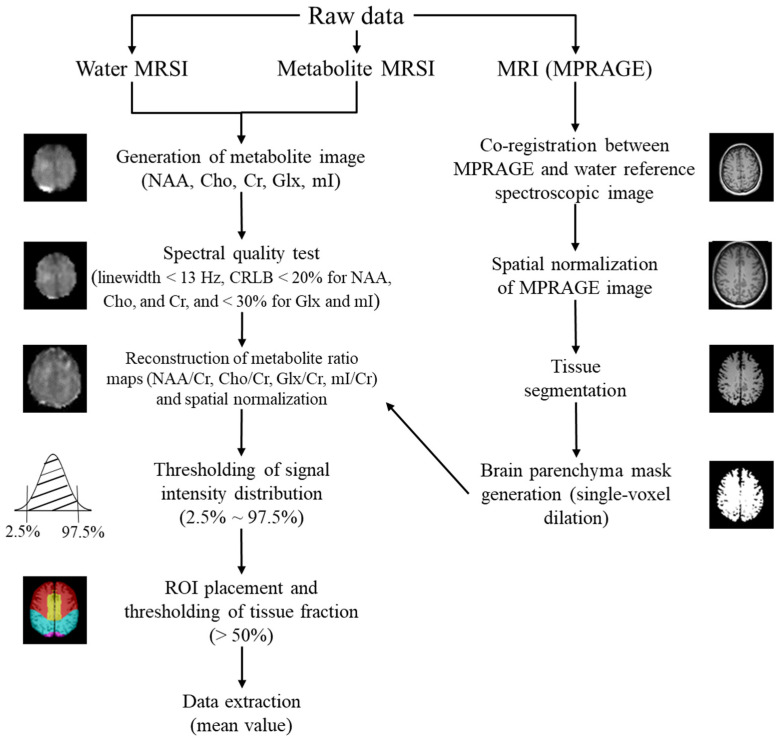
Scheme summarizing the processing steps (Abbreviations: CRLB = Cramer-Rao lower bound, MRSI = Magnetic resonance spectroscopic imaging, MPRAGE = T1-weighted 3D magnetization-prepared rapid acquisition gradient echo, NAA = N-acetylaspartate, Cho = choline, Cr = creatine, Glx = glutamate + glutamine, mI = myoinositol).

The processing steps are summarized in Figure 4. The steps closely followed the report by Maudsley et al. [7], and included data resampling, spatial reconstruction, B0 correction, spatial registration, brain and scalp mask formation for lipid k-space extrapolation, spectral fitting, and signal normalization. The major metabolite maps, i.e., NAA, Cho, Cr, Glx, and mI, representing their peak areas, were reconstructed from the WB-MRSI raw data; the reconstruction relied on the default parameters of the Metabolic Imaging and Data Analysis System (MIDAS) software version 2.35 (the University of Miami, Miami, FL, USA), which runs on IDL version 8.4.1 (Exelis Visual Information Solutions, Boulder, CO, USA) [7]. Spectral analysis was performed with the MIDAS FITT program, which carries out automated spectral fitting with parametric modeling based on prior knowledge of the metabolite resonances [54]. As a measure of spectral quality control, voxels were excluded if the spectral fitting reported a linewidth (i.e., full width at half maximum of the metabolic peak’s height) greater than 13 Hz and Cramer-Rao lower bound greater than 20% for NAA, Cho, and Cr and 30% for Glx and mI [55,56]. Signal normalization of the reconstructed metabolite maps was based on the tissue water signal derived from the interleaved water-reference MRSI. The signal normalization procedure used tissue water as an internal reference, which has been widely used for single-voxel MRS measurements and also applied to MRSI. Knowledge of the tissue water distribution was obtained by convolution of the MRI-derived tissue segmentations to the spatial response function and by using the calculation of the water content for gray matter and white matter, which was calculated with the PDWI. This procedure then derived a 100% water-equivalent reference image that corrected for the variable receiver sensitivity function and normalized the metabolite images. The resultant individual metabolite images therefore represent the metabolite signal obtained following spectral fitting relative to a reference signal equivalent to that of 100% water at the same voxel location. The signal normalization procedure included an estimate on the water T1. The metabolite ratio maps, i.e., NAA/Cr, Cho/Cr, Glx/Cr, and mI/Cr, were then reconstructed from the corresponding metabolite maps, using ImageJ software version 1.51 (National Institutes of Health, Bethesda, MD, USA) [57]. These metabolite ratio maps were used in further data analyses.

The reconstruction of the metabolite ratio maps was followed by the spatial normalization of these maps and the corresponding MPRAGE images by using the default parameters of Statistical Parametric Mapping 12 (SPM12) software (Wellcome Trust Centre for Neuroimaging, London, UK), which runs on MATLAB version 7.9.0 R2009b (The MathWorks, Natick, MA, USA). Voxels other than the brain tissue were removed by applying a brain parenchyma mask, which was formed by segmenting the brain tissue (gray and white matter) voxels from the corresponding spatially normalized MPRAGE images (SPM12) and dilating for a single voxel to limit partial volume errors (ImageJ). Those voxels with too low or too high signal intensity were also discarded, as these voxels were considered to be affected by magnetic field inhomogeneity and imperfect magnetic field shimming [9]. This process was achieved by discarding voxels with signal intensity below 2.5% and above 97.5% of each map’s total signal intensity distribution (ImageJ).

**Figure 5:**
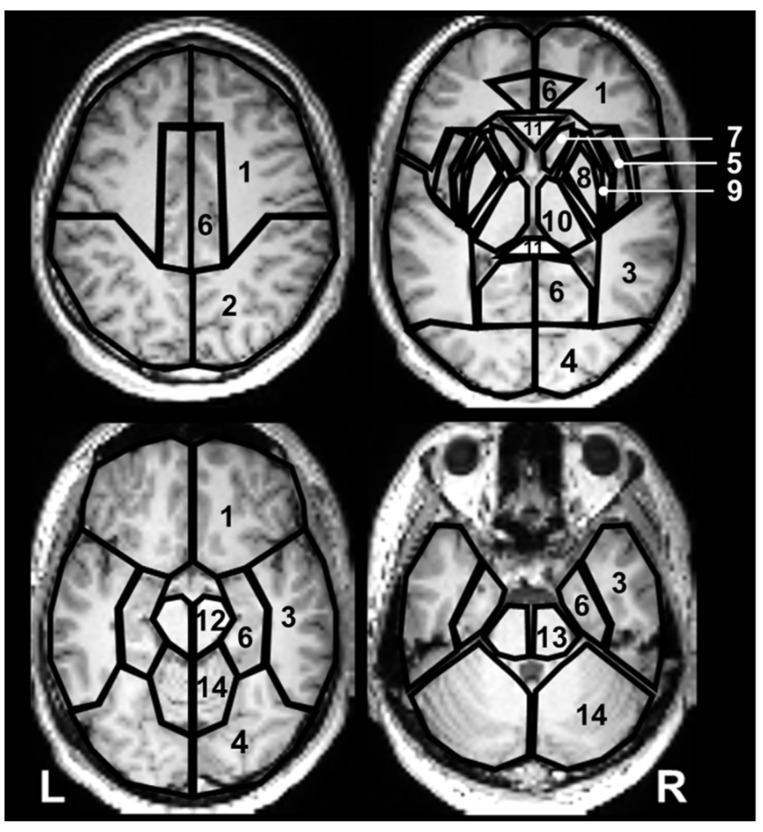
The outline of the regions of interest (ROIs) overlaid on the axial sections of normalized T1-weighted 3D magnetization-prepared rapid acquisition gradient echo (MPRAGE) images of a participant. Each ROI is placed on the either side of the frontal lobe (1); parietal lobe (2); temporal lobe (3); occipital lobe (4); insular lobe (5); limbic lobe (6); caudate (7) and lentiform nuclei (8); claustrum (9); thalamus (10); sublobar white matter (11) containing corpus callosum, external and internal capsules, and periventricular white matter; midbrain (12); pons (13); and cerebellar hemispheres (14).

Twenty-eight anatomical ROIs, which covered the entire brain, were selected from the WFU PickAtlas toolbox version 3.0.5 (Wake Forest University School of Medicine, Winston-Salem, NC, USA) [58] and applied on the resulting metabolite ratio maps. The exact anatomical location of each ROI is given in Figure 5. The mean metabolite ratios, including tissue-specific ratios, were then extracted for each ROI. Further evaluation of metabolite ratios’ distribution was limited to ROIs containing at least 50% of brain tissue; the evaluation used MRIcron version 1.0 (University of South Carolina, Columbia, SC, USA) [59]. This brain tissue fraction was chosen so as to include as many voxels as possible in the ROI while maximizing the sample size for each anatomical location. The gray and white matter volume ratio was recorded for each ROI.

### 4.4. Statistical Analysis

Unless otherwise specified, statistical analyses were performed by including both sides and sex groups to increase the number of voxels in each location and limit the number of comparisons (i.e., to limit type I error). Anatomical locations with the number of ROIs suitable for evaluation was less than ten (i.e., caudate nucleus, midbrain, pons, and cerebellar hemispheres) were discarded from further analysis, leaving 20 regions for evaluation.

The variations in metabolite ratios among anatomical regions were evaluated with repeated measures analysis of variance (ANOVA) and post hoc Bonferroni tests. The variations in metabolite ratios between laterality and in metabolite ratios between the two sides and sex groups were evaluated with two-sample t-tests and paired t-tests. The age-related changes in the regional metabolite ratios and their correlations were evaluated with Pearson’s product-moment correlation analyses. Age, BMI, sex, and the brain’s side were controlled in all analyses whenever appropriate, because these factors were reported to alter the metabolite concentrations [12,27]. *p* < 0.05 after correction for multiple comparisons was considered statistically significant. The difference in regional metabolite ratios between supine and prone positions, as evaluated by Bland–Altman analysis, was used to determine if scan conditions affected laterality in regional metabolite ratios. All statistical analyses were performed with Statistical Package for the Social Sciences (SPSS) software version 22 (IBM, New York, NY, USA).

## 5. Conclusions

This study evaluated the influence of anatomical location, sex, and age on the major metabolite ratios and their relationship by using short TE WB-MRSI. The metabolite ratios vary with anatomical location, sex, and age. Laterality in the regional metabolite ratios also exists. The interpretation of the regional metabolite ratios in a disease state in a clinical setting will require normative reference values. The results of this study may serve as a source for direct numerical comparison or for generating z-score maps. Finally, the major metabolite ratios are related, suggesting interactions among neural structures or neurotransmitters.

## Figures and Tables

**Table 1:** The mean regional metabolite ratios and their standard deviation (SD) for each side of the brain and sex.

Metabolite Ratio		Frontal Lobe	Parietal Lobe	Temporal Lobe	Occipital Lobe	Insula	Limbic Lobe	Claustrum	Lentiform Nucleus	Thalamus	Sublobar White Matter
NAA/Cr	Left	1.140 ± 0.086 *	1.188 ± 0.093 *	1.221 ± 0.085 *	1.240 ± 0.061	1.165 ± 0.095	1.173 ± 0.092	1.040 ± 0.093	1.021 ± 0.076	1.096 ± 0.08	1.212 ± 0.071
	Right	1.123 ± 0.077 *	1.163 ± 0.079 *	1.166 ± 0.086 *	1.232 ± 0.070	1.158 ± 0.099	1.175 ± 0.085	1.034 ± 0.110	1.043 ± 0.071	1.068 ± 0.087	1.204 ± 0.083
	Women	1.122 ± 0.069	1.173 ± 0.064	1.198 ± 0.071	1.226 ± 0.080	1.158 ± 0.095	1.164 ± 0.069	1.019 ± 0.111	1.015 ± 0.096	1.063 ± 0.105	1.208 ± 0.087
	Men	1.147 ± 0.069	1.181 ± 0.062	1.198 ± 0.072	1.214 ± 0.076	1.157 ± 0.093	1.182 ± 0.066	1.052 ± 0.111	1.037 ± 0.102	1.082 ± 0.104	1.194 ± 0.088
Cho/Cr	Left	0.218 ± 0.022	0.184 ± 0.020 *	0.207 ± 0.017 *	0.166 ± 0.015 *	0.230 ± 0.022 *	0.234 ± 0.026 *	0.246 ± 0.022	0.217 ± 0.020	0.239 ± 0.022	0.253 ± 0.020 *
	Right	0.217 ± 0.021	0.193 ± 0.020 *	0.219 ± 0.018 *	0.178 ± 0.016 *	0.234 ± 0.021 *	0.239 ± 0.025 *	0.248 ± 0.024	0.216 ± 0.019	0.245 ± 0.023	0.257 ± 0.020 *
	Women	0.207 ± 0.025 ^	0.185 ± 0.021	0.202 ± 0.020 ^	0.167 ± 0.018	0.223 ± 0.026 ^	0.229 ± 0.029	0.236 ± 0.033	0.214 ± 0.030	0.237 ± 0.028	0.250 ± 0.025
	Men	0.227 ± 0.025 ^	0.193 ± 0.021	0.220 ± 0.020 ^	0.176 ± 0.017	0.242 ± 0.026 ^	0.244 ± 0.027	0.255 ± 0.033	0.227 ± 0.032	0.249 ± 0.028	0.262 ± 0.025
Glx/Cr	Left	0.832 ± 0.075 *	0.834 ± 0.096	0.875 ± 0.061 *	0.783 ± 0.071	0.845 ± 0.086	0.843 ± 0.067 *	0.865 ± 0.100 *	0.770 ± 0.103	0.752 ± 0.112	0.785 ± 0.054 *
	Right	0.783 ± 0.070 *	0.807 ± 0.068	0.810 ± 0.054 *	0.778 ± 0.066	0.813 ± 0.083	0.808 ± 0.085 *	0.806 ± 0.114 *	0.717 ± 0.123	0.738 ± 0.086	0.747 ± 0.055 *
	Women	0.816 ± 0.086	0.825 ± 0.082	0.859 ± 0.071	0.759 ± 0.085	0.828 ± 0.111	0.842 ± 0.087	0.829 ± 0.145	0.714 ± 0.151	0.726 ± 0.122	0.771 ± 0.071
	Men	0.804 ± 0.086	0.813 ± 0.079	0.822 ± 0.072	0.783 ± 0.080	0.813 ± 0.108	0.810 ± 0.084	0.827 ± 0.146	0.753 ± 0.153	0.759 ± 0.120	0.751 ± 0.071
mI/Cr	Left	0.790 ± 0.082	0.723 ± 0.070	0.775 ± 0.055	0.661 ± 0.089	0.781 ± 0.088	0.834 ± 0.086	0.785 ± 0.102	0.678 ± 0.084	0.733 ± 0.150	0.848 ± 0.082
	Right	0.781 ± 0.074	0.736 ± 0.066	0.785 ± 0.051	0.680 ± 0.098	0.791 ± 0.066	0.820 ± 0.088	0.799 ± 0.092	0.673 ± 0.071	0.770 ± 0.138	0.842 ± 0.086
	Women	0.785 ± 0.085	0.700 ± 0.078 ^	0.770 ± 0.063	0.605 ± 0.110 ^	0.784 ± 0.091	0.817 ± 0.096	0.769 ± 0.131	0.629 ± 0.112 ^	0.772 ± 0.179	0.822 ± 0.101
	Men	0.782 ± 0.085	0.750 ± 0.076 ^	0.785 ± 0.062	0.709 ± 0.106 ^	0.802 ± 0.090	0.838 ± 0.092	0.826 ± 0.132	0.757 ± 0.115 ^	0.754 ± 0.178	0.865 ± 0.102

Note: * and ^ indicate statistical significance between the two sides and sex groups, respectively (*p* < 0.05). NAA = N-acetylaspartate, Cho = choline, Cr = creatine, Glx = glutamate + glutamine, mI = myoinositol.

**Table 2:** The relationship between major metabolite ratios of each anatomical location and age.

Metabolite Ratio		Frontal Lobe	Parietal Lobe	Temporal Lobe	Occipital Lobe	Insula	Limbic Lobe	Claustrum	Lentiform Nucleus	Thalamus	Sublobar White Matter
NAA/Cr	All	**−0.741**	**−0.791**	**−0.819**	**−0.668**	**−0.605**	**−0.780**	**−0.512**	−0.359	**−0.471**	**−0.568**
	Left	**−0.757**	**−0.821**	**−0.820**	**−0.672**	**−0.574**	**−0.783**	−0.502	−0.421	−0.111	−0.429
	Right	**−0.719**	**−0.766**	**−0.888**	−0.620	**−0.628**	**−0.779**	−0.528	−0.326	**−0.644**	**−0.639**
	Women	**−0.794**	**−0.783**	**−0.832**	−0.636	−0.437	**−0.834**	−0.329	−0.266	−0.342	−0.484
	Men	**−0.757**	**−0.817**	**−0.839**	**−0.696**	**−0.686**	**−0.776**	**−0.637**	−0.450	**−0.587**	**−0.677**
Cho/Cr	All	0.051	0.214	0.234	0.195	0.218	0.245	0.052	0.122	0.116	0.154
	Left	−0.008	0.123	0.348	0.241	0.176	0.181	0.052	0.252	0.108	0.128
	Right	0.106	0.307	0.062	0.225	0.229	0.293	0.049	0.033	0.099	0.156
	Women	−0.542	−0.313	−0.152	0.318	−0.249	−0.402	−0.313	−0.287	−0.054	−0.148
	Men	0.453	**0.494**	0.527	0.276	0.476	**0.510**	0.273	0.662	0.331	0.501
Glx/Cr	All	−0.262	**−0.532**	−0.262	−0.259	−0.143	**−0.422**	−0.023	0.004	−0.092	−0.248
	Left	−0.196	**−0.577**	−0.293	−0.427	0.094	−0.480	0.144	−0.068	−0.257	0.017
	Right	−0.316	−0.503	−0.243	0.139	−0.291	−0.364	−0.124	0.084	0.122	−0.360
	Women	0.001	−0.319	−0.051	0.035	−0.102	−0.114	0.083	0.296	0.125	0.102
	Men	−0.461	**−0.634**	−0.510	−0.360	−0.186	**−0.588**	−0.091	−0.207	−0.195	**−0.646**
mI/Cr	All	**0.417**	0.140	0.380	−0.269	**0.452**	**0.385**	0.316	−0.158	0.298	0.145
	Left	0.428	0.055	0.414	−0.185	0.310	0.431	0.426	−0.394	0.314	0.164
	Right	0.417	0.232	0.357	−0.354	**0.575**	0.355	0.236	0.177	0.270	0.136
	Women	0.496	0.306	0.105	−0.030	0.401	0.461	0.356	0.305	0.246	0.392
	Men	0.503	0.083	0.566	−0.175	**0.543**	0.412	0.450	0.054	0.443	0.188

Note: numbers in bold indicate statistical significance (corrected *p* < 0.05). NAA = N-acetylaspartate, Cho = choline, Cr = creatine, Glx = glutamate + glutamine, mI = myoinositol.

**Table 3:** Details about the participants.

Sex		Total Number (Right-Handers)	Mean Age ± SD (Range)	Mean Years of Education ± SD (Range)	Mean BMI ± SD
All		38 (30)	35.11 ± 11.97 (22–68) years	17.88 ± 2.12 (16–22) years	22.76 ± 2.92
	Women	17 (14)	34.29 ± 9.76 (22–52) years	18.00 ± 2.35 (16–22) years	20.90 ± 2.42
	Men	21 (16)	35.76 ± 13.71 (22–68) years	17.75 ± 1.98 (16–22) years	24.27 ± 2.39

Note: SD = standard deviation, BMI = body mass index.

## Data Availability

The data presented in this study are available on request from the corresponding author. The data are not publicly available due to patient confidentiality or ethical restrictions.

## Supplementary Materials

The following supporting information can be downloaded at: https://www.mdpi.com/article/10.3390/metabo12060543/s1, Figure S1: The representative major metabolite ratio maps overlaid on water-reference spectroscopic images. The LUT scale indicates metabolite ratios; Figure S2: The Bland-Altman plots between the two head positions for (A) NAA)/Cr, (B) Cho)/Cr, (C) Glx/Cr, and (D) mI/Cr, in twenty anatomical regions. The solid line indicates the mean difference and the dash lines limit of agreement; Table S1: Tissue-specific mean regional metabolite ratios and their SD; Table S2: The mean regional metabolite ratios and their SD for right-handers; Table S3: The sex-specific regional metabolite ratios and their SD for each cerebral hemisphere.
