# Supplementary material for: The Distribution of Major Brain Metabolites in Normal Adults: Short Echo Time Whole-Brain MR Spectroscopic Imaging Findings
Source: Metabolites. 2022 Jun 14;12(6):543. doi: 10.3390/metabo12060543 (PMC9228869; doi:10.3390/metabo12060543)
Supplement: Supplementary file 1 [file metabolites-12-00543-s001.zip › Table S1.pdf]

Table S1. Tissue-specific mean regional metabolite ratios and their standard deviation (SD).

Metabolite ratio		Frontal lobe	Parietal lobe	Temporal lobe	Occipital lobe	Insula	Limbic lobe	Claustrum	Lentiform nucleus	Thalamus	Sublobar white matter
GM	NAA/Cr	1.080 ± 0.082	1.159 ± 0.085	1.155 ± 0.082	1.191 ± 0.078	1.157 ± 0.091	1.129 ± 0.080	1.034 ± 0.091	0.968 ± 0.076	1.048 ± 0.095	1.069 ± 0.076
	Cho/Cr	0.190 ± 0.017	0.169 ± 0.013	0.194 ± 0.015	0.169 ± 0.011	0.224 ± 0.019	0.212 ± 0.019	0.247 ± 0.025	0.212 ± 0.024	0.237 ± 0.026	0.243 ± 0.020
	Glx/Cr	0.855 ± 0.061	0.867 ± 0.080	0.884 ± 0.066	0.811 ± 0.075	0.841 ± 0.093	0.868 ± 0.056	0.869 ± 0.149	0.787 ± 0.134	0.776 ± 0.134	0.821 ± 0.093
	mI/Cr	0.779 ± 0.069	0.757 ± 0.052	0.782 ± 0.033	0.670 ± 0.057	0.788 ± 0.064	0.852 ± 0.051	0.784 ± 0.077	0.658 ± 0.084	0.776 ± 0.099	0.819 ± 0.052
WM	NAA/Cr	1.164 ± 0.087	1.210 ± 0.093	1.240 ± 0.093	1.234 ± 0.087	1.226 ± 0.093	1.210 ± 0.092	1.107 ± 0.094	1.113 ± 0.103	1.117 ± 0.090	1.219 ± 0.081
	Cho/Cr	0.217 ± 0.021	0.189 ± 0.016	0.222 ± 0.018	0.176 ± 0.012	0.232 ± 0.021	0.238 ± 0.022	0.246 ± 0.023	0.231 ± 0.026	0.234 ± 0.023	0.256 ± 0.020
	Glx/Cr	0.802 ± 0.069	0.841 ± 0.083	0.836 ± 0.074	0.786 ± 0.071	0.796 ± 0.074	0.839 ± 0.056	0.815 ± 0.109	0.777 ± 0.098	0.732 ± 0.113	0.763 ± 0.064
	mI/Cr	0.802 ± 0.067	0.783 ± 0.052	0.831 ± 0.036	0.675 ± 0.062	0.802 ± 0.063	0.867 ± 0.056	0.780 ± 0.081	0.739 ± 0.078	0.780 ± 0.089	0.854 ± 0.051

Note: GM = gray matter, WM = white matter, NAA = N-acetyl aspartate, Cho = choline, Cr = creatine, Glx = glutamate+glutamine, mI = myoinositol
