# Supplementary material for: The Distribution of Major Brain Metabolites in Normal Adults: Short Echo Time Whole-Brain MR Spectroscopic Imaging Findings
Source: Metabolites. 2022 Jun 14;12(6):543. doi: 10.3390/metabo12060543 (PMC9228869; doi:10.3390/metabo12060543)
Supplement: Supplementary file 1 [file metabolites-12-00543-s001.zip › Table S2.pdf]

Table S2. The mean regional metabolite ratios and their standard deviation (SD) for right-handers.

Metabolite ratio		Frontal lobe	Parietal lobe	Temporal lobe	Occipital lobe	Insula	Limbic lobe	Claustrum	Lentiform nucleus	Thalamus	Sublobar white matter
NAA/Cr	Left	1.135 ± 0.081	1.182 ± 0.088*	1.204 ± 0.086*	1.241 ± 0.063	1.152 ± 0.098	1.172 ± 0.091	1.022 ± 0.096	1.004 ± 0.056	1.088 ± 0.084	1.204 ± 0.069
	Right	1.120 ± 0.070	1.162 ± 0.077*	1.156 ± 0.092*	1.230 ± 0.066	1.138 ± 0.093	1.175 ± 0.083	1.002 ± 0.101	1.021 ± 0.071	1.055 ± 0.090	1.192 ± 0.082
Cho/Cr	Left	0.219 ± 0.022	0.185 ± 0.020*	0.207 ± 0.019	0.167 ± 0.012	0.232 ± 0.021	0.235 ± 0.024	0.245 ± 0.022	0.216 ± 0.023	0.241 ± 0.019	0.254 ± 0.019
	Right	0.219 ± 0.021	0.194 ± 0.021*	0.220 ± 0.020	0.180 ± 0.015	0.236 ± 0.019	0.242 ± 0.025	0.247 ± 0.021	0.216 ± 0.022	0.245 ± 0.023	0.259 ± 0.019
Glx/Cr	Left	0.823 ± 0.076*	0.822 ± 0.091	0.866 ± 0.063*	0.775 ± 0.070	0.844 ± 0.089	0.838 ± 0.071*	0.860 ± 0.093	0.759 ± 0.108	0.742 ± 0.106	0.788 ± 0.052*
	Right	0.772 ± 0.065*	0.795 ± 0.069	0.803 ± 0.059*	0.774 ± 0.061	0.798 ± 0.081	0.800 ± 0.083*	0.813 ± 0.124	0.729 ± 0.127	0.737 ± 0.080	0.739 ± 0.056*
mI/Cr	Left	0.789 ± 0.088	0.711 ± 0.069*	0.772 ± 0.061	0.642 ± 0.089	0.781 ± 0.094	0.830 ± 0.092	0.775 ± 0.102	0.680 ± 0.091	0.736 ± 0.158	0.857 ± 0.085
	Right	0.779 ± 0.079	0.731 ± 0.058*	0.782 ± 0.058	0.667 ± 0.108	0.791 ± 0.063	0.819 ± 0.094	0.800 ± 0.083	0.683 ± 0.073	0.767 ± 0.138	0.851 ± 0.093

Note: * indicates statistical significance between the two sides ($P < 0.05$). NAA = N-acetyl aspartate, Cho = choline, Cr = creatine, Glx = glutamate+glutamine, mI = myoinositol
