# Supplementary material for: The Distribution of Major Brain Metabolites in Normal Adults: Short Echo Time Whole-Brain MR Spectroscopic Imaging Findings
Source: Metabolites. 2022 Jun 14;12(6):543. doi: 10.3390/metabo12060543 (PMC9228869; doi:10.3390/metabo12060543)
Supplement: Supplementary file 1 [file metabolites-12-00543-s001.zip › Table S3.pdf]

Table S3. The sex-specific regional metabolite ratios and their standard deviation (SD) for each cerebral hemisphere.

Location			Frontal lobe	Parietal lobe	Temporal lobe	Occipital lobe	Insula	Limbic lobe	Claustrum	Lentiform nucleus	Thalamus	Sublobar white matter
NAA/Cr	Left	Women	1.148 ± 0.072	1.194 ± 0.063	1.229 ± 0.071	1.217 ± 0.082	1.178 ± 0.099	1.164 ± 0.072	1.037 ± 0.101	1.008 ± 0.102	1.091 ± 0.100	1.214 ± 0.089
		Men	1.150 ± 0.072	1.190 ± 0.062	1.207 ± 0.071	1.207 ± 0.081	1.152 ± 0.099	1.180 ± 0.069	1.045 ± 0.101	1.018 ± 0.110	1.101 ± 0.101	1.210 ± 0.093
	Right	Women	1.100 ± 0.068	1.150 ± 0.065	1.147 ± 0.075	1.242 ± 0.084	1.139 ± 0.095	1.163 ± 0.068	1.003 ± 0.122	1.016 ± 0.097	1.033 ± 0.105	1.199 ± 0.089
		Men	1.145 ± 0.067	1.173 ± 0.062	1.188 ± 0.077	1.226 ± 0.075	1.161 ± 0.091	1.183 ± 0.066	1.057 ± 0.122	1.058 ± 0.101	1.067 ± 0.103	1.185 ± 0.088
Cho/Cr	Left	Women	0.208 ± 0.025	0.183 ± 0.022	0.196 ± 0.020	0.163 ± 0.017	0.219 ± 0.026	0.224 ± 0.029	0.238 ± 0.032	0.220 ± 0.027	0.231 ± 0.027	0.248 ± 0.026
		Men	0.226 ± 0.025	0.187 ± 0.021	0.215 ± 0.020	0.171 ± 0.016	0.240 ± 0.026	0.243 ± 0.028	0.250 ± 0.032	0.216 ± 0.030	0.248 ± 0.027	0.258 ± 0.027
	Right	Women	0.207 ± 0.026	0.186 ± 0.022	0.209 ± 0.021	0.172 ± 0.022	0.227 ± 0.027	0.235 ± 0.029	0.235 ± 0.036	0.209 ± 0.033	0.242 ± 0.029	0.252 ± 0.025
		Men	0.229 ± 0.026	0.198 ± 0.021	0.225 ± 0.021	0.182 ± 0.020	0.244 ± 0.026	0.245 ± 0.028	0.260 ± 0.035	0.236 ± 0.035	0.251 ± 0.029	0.266 ± 0.025
Glx/Cr	Left	Women	0.841 ± 0.090	0.827 ± 0.087	0.893 ± 0.074	0.753 ± 0.077	0.860 ± 0.109	0.859 ± 0.075	0.883 ± 0.135	0.736 ± 0.160	0.738 ± 0.139	0.797 ± 0.072
		Men	0.827 ± 0.090	0.833 ± 0.085	0.838 ± 0.074	0.786 ± 0.075	0.833 ± 0.109	0.830 ± 0.072	0.836 ± 0.138	0.774 ± 0.165	0.765 ± 0.139	0.771 ± 0.074
	Right	Women	0.793 ± 0.086	0.829 ± 0.073	0.812 ± 0.073	0.772 ± 0.100	0.798 ± 0.112	0.826 ± 0.101	0.779 ± 0.153	0.693 ± 0.154	0.719 ± 0.106	0.745 ± 0.072
		Men	0.783 ± 0.086	0.790 ± 0.070	0.805 ± 0.077	0.782 ± 0.086	0.794 ± 0.108	0.790 ± 0.097	0.818 ± 0.153	0.743 ± 0.154	0.749 ± 0.104	0.735 ± 0.071
mI/Cr	Left	Women	0.795 ± 0.091	0.694 ± 0.080	0.775 ± 0.063	0.580 ± 0.095*	0.785 ± 0.106	0.828 ± 0.098	0.736 ± 0.113*	0.583 ± 0.145*	0.772 ± 0.182	0.831 ± 0.103
		Men	0.779 ± 0.090	0.742 ± 0.078	0.777 ± 0.062	0.713 ± 0.094*	0.782 ± 0.106	0.839 ± 0.094	0.852 ± 0.114*	0.836 ± 0.153*	0.692 ± 0.184	0.867 ± 0.106
	Right	Women	0.776 ± 0.083	0.708 ± 0.079	0.762 ± 0.066	0.652 ± 0.136	0.784 ± 0.080	0.805 ± 0.098	0.798 ± 0.148	0.663 ± 0.070	0.768 ± 0.178	0.814 ± 0.105
		Men	0.785 ± 0.083	0.758 ± 0.075	0.797 ± 0.066	0.699 ± 0.126	0.819 ± 0.077	0.836 ± 0.094	0.804 ± 0.148	0.703 ± 0.070	0.810 ± 0.174	0.865 ± 0.104

Note: * indicates statistical significance between the gender groups ($P < 0.05$). NAA = N-acetyl aspartate, Cho = choline, Cr = creatine, Glx = glutamate+glutamine, mI = myoinositol
